# Structure of the full-length TRPV2 channel by cryo-EM

**DOI:** 10.1038/ncomms11130

**Published:** 2016-03-29

**Authors:** Kevin W. Huynh, Matthew R. Cohen, Jiansen Jiang, Amrita Samanta, David T. Lodowski, Z. Hong Zhou, Vera Y. Moiseenkova-Bell

**Affiliations:** 1 Department of Pharmacology, School of Medicine, Case Western Reserve University, 10900 Euclid Avenue, Wood Building, W151D, Cleveland, Ohio 44106, USA; 2 Department of Physiology and Biophysics, School of Medicine, Case Western Reserve University, Cleveland, Ohio 44106, USA; 3 Department of Microbiology, Immunology and Molecular Genetics, University of California, Los Angeles, California 90095, USA; 4 California NanoSystems Institute, University of California, Los Angeles, California 90095, USA; 5 Department of Nutrition, School of Medicine, Case Western Reserve University, Cleveland, Ohio 44106, USA

## Abstract

Transient receptor potential (TRP) proteins form a superfamily Ca^2+^-permeable cation channels regulated by a range of chemical and physical stimuli. Structural analysis of a ‘minimal' TRP vanilloid subtype 1 (TRPV1) elucidated a mechanism of channel activation by agonists through changes in its outer pore region. Though homologous to TRPV1, other TRPV channels (TRPV2–6) are insensitive to TRPV1 activators including heat and vanilloids. To further understand the structural basis of TRPV channel function, we determined the structure of full-length TRPV2 at ∼5 Å resolution by cryo-electron microscopy. Like TRPV1, TRPV2 contains two constrictions, one each in the pore-forming upper and lower gates. The agonist-free full-length TRPV2 has wider upper and lower gates compared with closed and agonist-activated TRPV1. We propose these newly revealed TRPV2 structural features contribute to diversity of TRPV channels.

## Abstract (web summary)

Transient receptor potential (TRP) proteins are Ca^2+^-permeable cation channels activated by a range of chemical and physical stimuli. Here the authors describe a cryo-EM structure of the full-length TRPV2 channel that provides insight into the regulation of the TRPV subfamily of channels.

## Graphical abstract

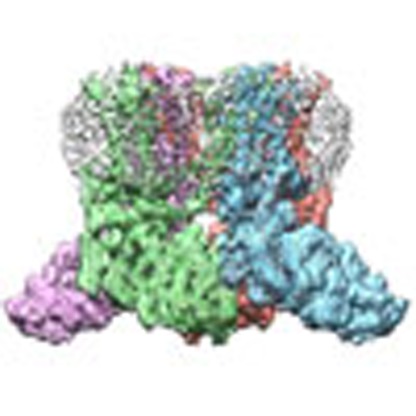

Local and global Ca^2+^ transients tightly regulate diverse cellular events such as gene transcription, cell motility, muscle contraction and sensory transduction^1^. Twenty-eight mammalian TRP homologues from six subfamilies (TRPV, TRPM, TRPC, TRPA, TRPP and TRPML) are essential Ca^2+^-permeable channels, many of which are activated by chemical agonists^2^. The TRPV subfamily consists of six members (TRPV1–6). TRPV1 is the most commonly studied TRPV channel and is activated by noxious heat, and endogenous and exogenous vanilloids^3^,^4^. Structures of a ‘minimal' rat TRPV1 construct in ligand-free and -bound states determined by cryo-electron microscopy (cryo-EM) have provided tremendous insight into the conformational dynamics of channel opening on ligand activation^5^,^6^. The remaining members of the TRPV subfamily (TRPV2–6) share 30–50% sequence similarity with TRPV1 (ref. 7); nevertheless they are vanilloid insensitive and play a minimal role in temperature sensation^8^,^9^,^10^,^11^, suggesting that ‘minimal' TRPV1 is only partly representative of the other TRPV subfamily members.

TRPV2 is a member of the TRPV subfamily and shares ∼50% sequence identity with TRPV1 (refs 7, 12). Nevertheless, it differs from TRPV1 in its overall cellular function^7^. While heterologously expressed and endogenous TRPV1 localizes to the plasma membrane (Supplementary Fig. 1a,c) and is activated by noxious heat and endogenous and exogenous vanilloids^3^,^4^, heterologously expressed TRPV2 predominantly localizes to intracellular membranes^13^ (Supplementary Fig. 1b,c). In addition, TRPV2 does not respond to heat or vanilloids *in vivo*^8^,^12^,^13^,^14^. In embryonic DRG neurons and other neuronal cell lines, endogenous TRPV2 shows a punctate distribution within the soma and axons and colocolizes with Rab7, a late endosomal marker, where it promotes neuronal outgrowth downstream of neurotrophin signalling^14^.

In heterologous expression systems, TRPV2 activity was shown to be increased by exogenous small molecules such as 2-aminoethoxydiphenyl borate (2-APB) and probenecid, and blocked by ruthenium red and Gd^3^,^7^,^15^. We previously found that recombinant TRPV2 reconstituted in artificial liposomes displayed current in the presence of probenecid that was blocked by Gd^16^. However, physiological regulators of TRPV2 and other TRPV channels remain unknown^7^. It has been suggested that post-translational modifications, lipid–protein interactions and protein–protein interactions may regulate the function of these channels^2^. Specifically, phosphatidylinositol 4,5-bisphosphate (PIP_2_) levels in the membrane may modulate TRPV2 activity^17^, and direct phosphorylation of TRPV2 by extracellular-signal-regulated kinases has been shown to regulate TRPV2 expression and activity^14^. A detailed structural analysis of TRPV2 may provide deeper insight into the cellular function of TRPV2, which has been elusive since its discovery^7^,^12^,^18^.

Here we report the structure of full-length rat TRPV2 at ∼5 Å resolution determined by cryo-EM single particle reconstruction. Our structure reveals a pore region with two constrictions or gates as observed in other TRP channels^5^,^6^,^19^. In the absence of ligands, TRPV2 displays wider upper and lower gates compared with closed and agonist-activated TRPV1. These results further our understanding of the diverse functional regulation of the TRPV subfamily of channels.

## Results

### Structural determination of full-length TRPV2

**Figure 1:**
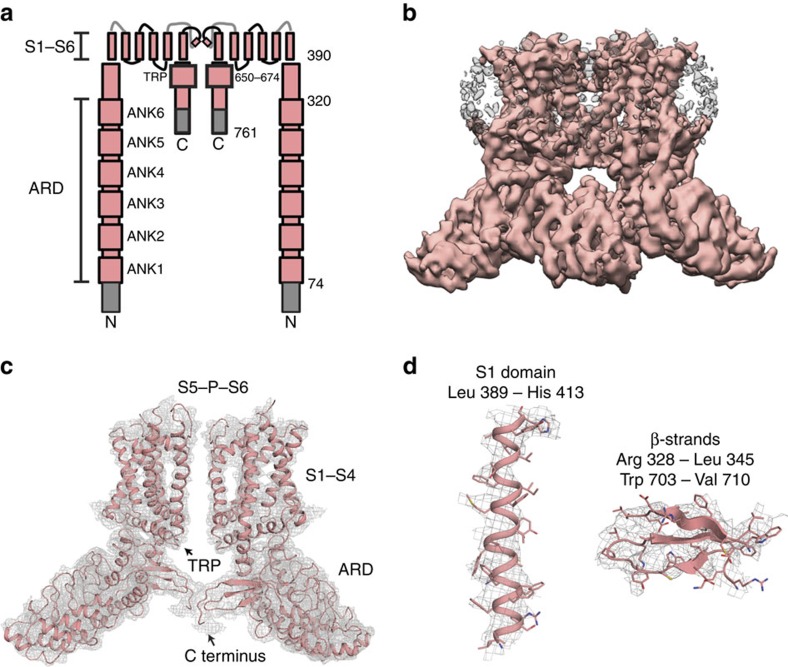
Single particle cryo-EM analysis of full-length TRPV2. (**a**) Schematic depiction of full-length rat TRPV2 shown as a dimer with its ankyrin repeat domain (ARD), S1–S4 helices, S5–P–S6 pore domain and C terminus. Missing densities in the corresponding full-length TRPV2 cryo-EM map are depicted in grey. (**b**) Final full-length TRPV2 cryo-EM map. (**c**) The TRPV2 atomic model is superimposed onto two subunits of the tetrameric full-length TRPV2 cryo-EM map. (**d**) Superimposition of representative regions of the full-length TRPV2 cryo-EM map (mesh) with the atomic model (ribbons and sticks), including α helix and β strand.

Our previous cryo-EM structure of full-length rat TRPV2 provided a structure at 13.6 Å, which revealed the general architecture of TRPV subfamily members^16^. This moderate resolution structure, however, did not yield a detailed understanding of the TRPV2 channel structure and function^16^. Recent progress in direct electron-counting technology^20^ and statistical image processing^21^ has allowed us to determine the structure of TRPV2 in the absence of ligand (apo-TRPV2) to ∼5 Å, resolving strands in β sheets and some bulky side chains of amino acid residues (Fig. 1, Supplementary Movies and Supplementary Figs 2–5). Individual particles are apparent in the micrographs (Supplementary Fig. 2a). Details of the full-length channel are visible in the two-dimensional (2D) class averages (Supplementary Fig. 2b) and the final 3D reconstructed map shows a tetrameric assembly (Supplementary Fig. 2c,d). Based on the cryo-EM map, we are able to build models for all transmembrane (TM) helices (S1–S6), the pore helix, the selectivity filter, the N-terminal linker and a portion of the C terminus of the channel using the previously published TRPV1 atomic models^5^,^6^ as homology models (Fig. 1, Supplementary Fig. 5 and see Methods). All six N-terminal ankyrin repeats of the ankyrin repeat domain (ARD) are clearly resolved, suggesting that the ARD is a rigid, stable domain in full-length TRPV2 (Fig. 1; Supplementary Fig. 5a). Despite the expression of full-length TRPV2, density accounting for the first 73 residues of the N terminus (Met 1–Asp 73), the S1–S2 linker (Ile 422–Gly 430), the pore turret (Glu 561–Leu 594) and the last 46 residues of the C terminus (Glu 716–Pro 761) are not apparent in the cryo-EM map (Fig. 1a; grey), suggesting that these regions are flexible or disordered.

### Pore architecture of full-length TRPV2

**Figure 2:**
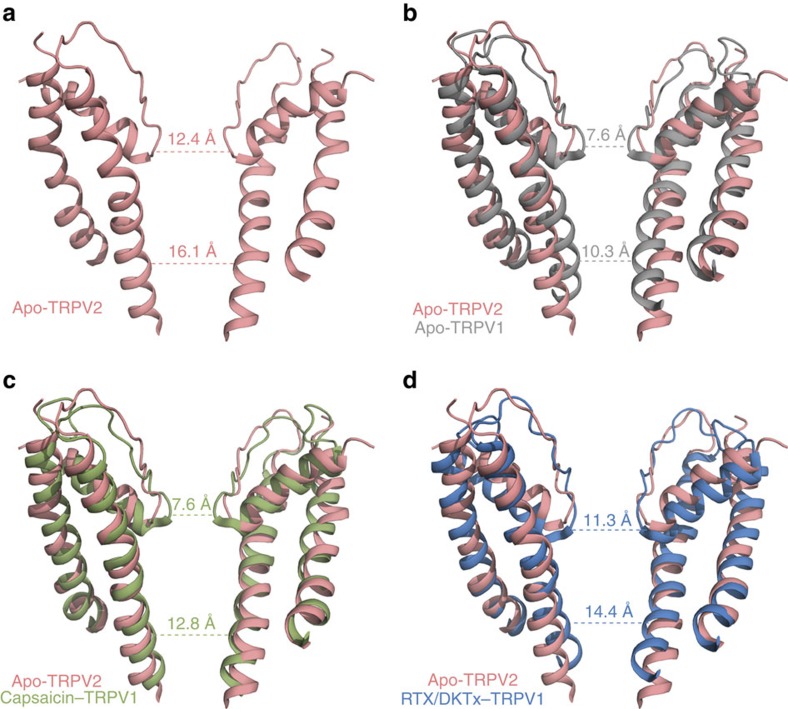
Analysis of the TRPV2 ion permeation pathway. (**a**) Atomic model of the S5–P–S6 region of apo-TRPV2. Two subunits are shown for clarity. The dotted lines show the Cα–Cα distances in the apo-TRPV2 structure, which represent the narrowest points in the upper and lower gates. (**b**–**d**) Atomic model of the S5–P–S6 region of apo-TRPV2 (salmon) superimposed onto the (**b**) apo-TRPV1 structure (grey, PDB: 3J5P), (**c**) capsaicin–TRPV1 structure (green, PDB: 3J5R) and (**d**) RTX/DkTx–TRPV1 structure (blue, PDB: 3J5Q). The dotted lines show the Cα–Cα distances at the narrowest points in the upper and lower gates of the TRPV1 structures.

The general architecture of apo-TRPV2 has been proposed to be similar to the structures of other TRP channels, as well as distantly related voltage- and ligand-gated channels^6^,^19^,^22^,^23^,^24^,^25^. The 2D class averages and the final 3D reconstructed map of TRPV2 unambiguously show a tetrameric assembly similar to TRPV1 (Fig. 1, Supplementary Fig. 2). Like TRPV1 and TRPA1, the pore of apo-TRPV2 consists of two constrictions or gates^5^,^6^,^19^: an upper gate resides at the selectivity filter in the outer pore region of the channel and a lower gate is comprised of the distal end of S6 (Fig. 2a). A more detailed structural analysis of the apo-TRPV2 pore domain unexpectedly revealed that the upper and lower constrictions are wider than the homologous sites of TRPV1 (Gly 643 and Ile 679) in the absence of ligand (apo-TRPV1) with distances of 12.4 Å and 16.1 Å when measured Cα–Cα at the upper and lower constrictions, respectively (Fig. 2a). The structure of apo-TRPV1 was proposed to comprise a state in which both the upper and lower gates are closed^6^ (Fig. 2b), whereas in the presence of the agonists resiniferatoxin (RTX) and double-knot toxin (RTX/DkTx–TRPV1) both gates are open^5^,^6^ (Fig. 2d). In addition, a structure of TRPV1 in the presence of capsaicin consists of a closed upper gate and partially open lower gate (Fig. 2c), and may represent an intermediate conformation of the channel^5^,^6^. The upper gate of apo-TRPV2 is wider than the closed upper gate of TRPV1 (apo- and capsaicin–TRPV1), as well as the open upper gate of TRPV1 (RTX/DkTx–TRPV1; Fig. 2b,c). In addition, the lower gate of apo-TRPV2 has a larger Cα–Cα distance compared with TRPV1 in all states, including the open lower gate in RTX/DkTx–TRPV1 (14.4 Å; Fig. 2b–d). These results suggest that the TRPV2 channel can accommodate partially hydrated Ca^2+^, Na^+^ and K^+^ ions^26^, as well as large organic cations^27^ in the apo state.

### Position of the ARD and pore helix in full-length TRPV2

**Figure 3:**
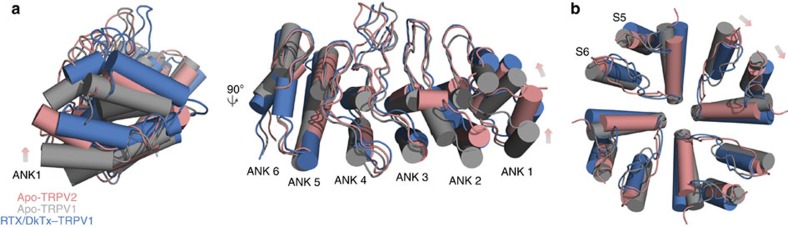
Comparison of the ankyrin repeat domain and outer pore region of TRPV2 and TRPV1. (**a**) Two representative views of the superimposed ARDs of apo-TRPV1 (grey), RTX/DkTx–TRPV1 (blue) and apo-TRPV2 (salmon). Arrows represent displacement of the apo-TRPV2 ARDs relative to apo-TRPV1. (**b**) Superimposed top–down views of outer pore regions of apo-TRPV1 (grey), RTX/DkTx–TRPV1 (blue) and apo-TRPV2 (salmon). Note the shift in position of the pore helix and S5 in the clockwise direction of both RTX/DkTx–TRPV1 and apo-TRPV2 relative to apo-TRPV1.

Molecular dynamics simulations based on closed and open structures of TRPV1 predicted that channel opening results from a sequence of conformational changes beginning in the intracellular ARDs, followed by allosteric changes in the upper gate then the lower gate, including a quaternary twist of the pore domain relative to the intracellular domain^28^. We therefore compared the position of the apo-TRPV2 ARD with apo-TRPV1 and RTX/DkTx–TRPV1 to gain further insights into the apo-TRPV2 structure (Fig. 3a). The upward shift of the ARD is apparent between apo-TRPV1 and RTX/DkTx–TRPV1 (Fig. 3a). Our comparison showed that position of the apo-TRPV2 ARD lies between apo-TRPV1 and RTX/DkTx–TRPV1 (Fig. 3a). Moreover, we compared the position of the pore helix of apo-TRPV2 relative to the central axis of the channel as it has been proposed that twist of the pore helix in the RTX/DkTx–TRPV1 structure contributes to opening of the upper gate (Fig. 3b)^5^. The pore helix of RTX/DkTx–TRPV1 shows a clockwise twist relative to apo-TRPV1 (Fig. 3b), while the position of the apo-TRPV2 pore helix again falls between apo-TRPV1 and RTX/DkTx–TRPV1 (Fig. 3b). In addition, the position of the apo-TRPV2 S5 helix resembles that of the RTX/DkTx–TRPV1 (Fig. 3b). Together these results suggest that in our cryo-EM structure of the apo-TRPV2 channel, the positions of the ARDs and the pore helix differ from both apo-TRPV1 and RTX/DkTx–TRPV1.

### Divergence in the outer pore region of full-length TRPV2

TRPV1 and TRPV2 share high-sequence identity throughout the S5, S6, pore helix and selectivity filter (Gly–Met–Gly–Asp/Glu). Nonetheless, TRPV1 and TRPV2 are non-selective cation channels with varying Ca^2+^ selectivity (*P*_Ca_^2+^/*P*_Na_^+^=10 for TRPV1 and *P*_Ca_^2+^/*P*_Na_^+^=3 for TRPV2)^7^. The observed structural divergence in the upper gates in the outer pore region between apo-TRPV1 and apo-TRPV2 (Fig. 2) may explain the differences in cation selectivity between the two channels. The differences in pore architecture observed in this analysis of apo-TRPV1 and apo-TRPV2 may be due to inherent structural differences between these two proteins^7^. Conversely, the TM domain of the proteins was stabilized in different manners during the purification process. While apo-TRPV1 and ligand-bound TRPV1 were stabilized in A8–35 amphipol^5^,^6^,^29^,^30^,^31^, TRPV2 was purified using the maltose neopentyl glycol (MNG) class of detergents^16^,^31^,^32^. This difference in the biochemical preparations may have enabled the TRPV preparations to sample different conformational states, resulting in the observed divergence in the channel architectures^29^,^30^,^31^.

**Figure 4:**
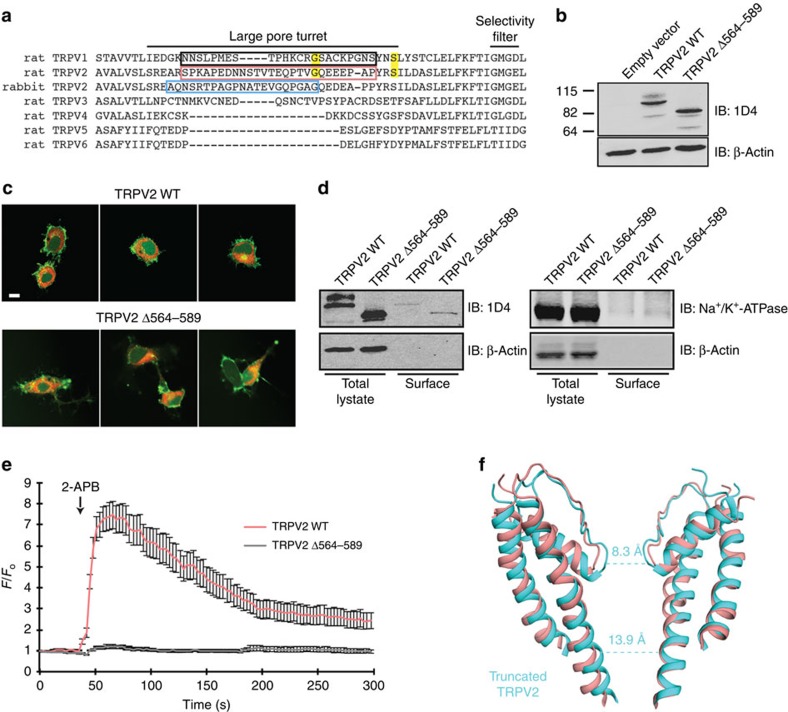
Large-pore turret may influence the TRPV2 ion permeation pathway. (**a**) Sequence alignment of the pore turret region of the TRPV family members. Black and cyan box highlight the truncated pore turret pore region in the ‘minimal' TRPV1 and truncated rabbit TRPV2 proteins used for structure determination, respectively. The salmon box highlights the corresponding region truncated in the TRPV2 protein (TRPV2 Δ564–589) for this analysis. (**b**) Western blot analysis of F11 cells transfected with empty vector, 1D4-tagged TRPV2 WT or 1D4-tagged TRPV2 Δ564–589 using indicated antibodies. (**c**) F11 cells were co-transfected with plasma membrane-GFP (GFP-labelled palmitoylation sequence from GAP43; green) and either 1D4-tagged TRPV2 WT or TRPV2 Δ564–589. Cells were fixed and immunostained using anti-1D4 antibody (red). Scale bar, 10 μm. (**d**) Surface biotinylation analysis of F11 cells transiently expressing either 1D4-tagged TRPV2 WT or TRPV2 Δ564–589. Proteins were visualized by western blot analysis using the indicated antibodies. (**e**) Intracellular calcium response of F11 cells transfected with TRPV2 WT (blue; *n*=50) or TRPV2 Δ564–589 (red; *n*=59) was recorded by Fluo4 fluorescence in response to 100 μM 2-APB. Cells were treated with 2-APB at the 45-s time point (indicated by arrow). Data represent the normalized mean Fluo4 intensity. (**f**) Atomic model of the S5–P–S6 region of truncated rabbit TRPV2 superimposed onto the same region of full-length rat TRPV2. Two subunits are shown for clarity. The dotted lines show the Cα–Cα distances in the truncated rabbit TRPV2 structure, which represents the upper and lower gates.

It is also possible that the truncations necessary for stabilization of ‘minimal subunit' TRPV1 led to alterations in the pore architecture of the TRPV1 structure. Specifically, a ∼30 amino acid loop connecting S5 with the pore helix, known as the pore turret (Fig. 4a), has been implicated in channel gating, large organic cation permeation and pore dilation^33^,^34^. Mutation of key residues within the TRPV1 pore turret (Gly 618 and Ser 629), which are conserved in TRPV2 (Gly 582 and Ser 592; Fig. 4a), altered the permeability of TRPV1 to large cations^33^, indicating that the pore turret contributes to expansion of the upper gate. Stabilization of TRPV1 for cryo-EM studies required deletion of the pore turret region^5^,^6^, while in the present work we employed full-length TRPV2 where the turret region is intact, although poorly resolved due to its flexibility. Homology modelling predicts that deletion of the turret region in the TRPV1 structures would alter the pore region architecture^34^, thus possibly accounting for some of the observed differences in pore architecture between TRPV1 and TRPV2 (Fig. 2). We found that truncation of the pore turret domain in TRPV2 (TRPV2 Δ564–589) at sites comparable to ‘minimal subunit' TRPV1 (ref. 6; Fig. 4a) had little effect on protein expression (Fig. 4b, Supplementary Fig. 9a), localization (Fig. 4c) and cell surface levels (Fig. 4d, Supplementary Fig. 9b) compared with TRPV2 WT. However, expression of TRPV2 Δ564–589 ablated the Ca^2+^ response to 2-APB, an exogenous TRPV2 activator^35^, compared with cells expressing TRPV2 WT (Fig. 4e).

While the current work was under review, a cryo-EM structure of a truncated rabbit TRPV2 construct appeared^36^. In addition to truncations at the N and C terminus, the rabbit TRPV2 construct used for cryo-EM analysis also lacked the pore turret domain^36^ (Fig. 4a). The outer pore region of the truncated rabbit TRPV2 assumed a conformation similar to that of the ‘minimal' rat apo-TRPV1 (refs 5, 6, 36), as expected due to the similarity in the channel truncations and biochemical approaches employed for stabilization in these studies^5^,^6^,^36^.

Overall structural differences between truncated rabbit TRPV2 and full-length rat TRPV2 are apparent in both cryo-EM densities and atomic models (Supplementary Fig. 6). Comparison of the pore region between truncated rabbit TRPV2 and full-length rat TRPV2 revealed that full-length rat TRPV2 contains wider upper and lower gates (Fig. 4f). Contrary to the Ca^2+^ result presented^36^, our data show that truncation of the pore turret of rat TRPV2 diminishes the response of the channel to 2-APB (Fig. 4e). The observed differences in the Ca^2+^ measurements between both truncated TRPV2 orthologues could be attributed to many different experimental variables. TRPV2 activation by 2-APB is known to be species dependent^37^,^38^; therefore the response of rat TRPV2 to 2-APB may be inherently different from that of rabbit TRPV2. A parallel characterization of how 2-APB interacts with rat versus rabbit TRPV2 may provide insights into how 2-APB activates TRPV2 orthologues.

### Lipid-binding sites in the full-length TRPV2 structure

Membrane lipids are known modulators of the TRPV channel function^5^,^36^,^39^. Truncated rabbit TRPV2 was purified in the presence of lipids/cholesterol hemisuccinate and densities for cholesterol molecule were proposed to occupy the crevice formed by the S1–S4 helical bundle above the TRP domain (Supplementary Fig. 7a). Moreover, weak densities that may represent lipid molecules were also observed in apo-TRPV1 and RTX/DkTx–TRPV1 (refs 5, 6, 36; Supplementary Fig. 7c,d). Based on these observations, it was suggested that lipid binding in this region of the channel could play a role in modulation of channel function^36^. We observe densities in a similar region similar to that in apo-TRPV1 and RTX/DkTx–TRPV1 (Supplementary Fig. 7c,d), which may represent lipid molecules that were co-purified with full-length rat TRPV2 (Supplementary Fig. 7b).

Another possible lipid density was observed in the truncated rabbit TRPV2 between S4, the S4–S5 linker and S6 of an adjacent subunit (Supplementary Fig. 8a) located in a similar position as the RTX molecule in the RTX/DkTx–TRPV1 structure (Supplementary Fig. 8b). In addition, a weak lipid density was observed in the apo-TRPV1 structure^5^,^6^,^36^ (Supplementary Fig. 8c). It was proposed that localization of the lipid molecules under physiological conditions in this region of TRPV1 and TRPV2 is conserved, and exchange of lipid molecules and lipophilic ligands lead to stabilization of the open state of the channels^36^. We did not observe a lipid density in this region of our structure of the full-length rat TRPV2 purified without added lipids (Supplementary Fig. 8d). Intriguingly, the positions of the S4 helix, S4–S5 linker and S6 of an adjacent subunit in the full-length rat TRPV2 are different from the positions in the truncated rabbit TRPV2 (Supplementary Fig. 8d). While in the full-length TRPV2, the S4 helix adopted conformation similar to that of apo-TRPV1 (Supplementary Fig. 8e), the S4 helix of truncated rabbit TRPV2 favours the position of RTX/DkTx–TRPV1 (Supplementary Fig. 8f). This observed structural difference suggests that interaction of this region of the TRPV channels with lipids (either physiological or added during purification) is dynamic and possibly affects channel function. The fact that we observed a wider pore for full-length rat TRPV2 without added lipid is consistent with the hypothesis that added lipid promotes a desensitized state for truncated rabbit TRPV2 (ref. 36). However, differences in the structures of full-length TRPV2 and truncated TRPV2 may also be due to a number of variables, including inherent structural differences between TRPV orthologues, deletions of N- and C-terminal amino acids in addition to truncation of the pore turret and differences in biochemical protein preparation for cryo-EM.

## Discussion

Our analysis reveals that in the absence of ligand, full-length rat TRPV2 contains wider upper and lower gates compared with the ‘minimal' rat TRPV1 and truncated rabbit TRPV2. TRPV2 is insensitive to TRPV1 activators such as vanilloids and heat *in vivo*^8^,^12^. To date, endogenous regulators of TRPV2 activity remain unclear^7^. The majority of TRPV subfamily members are proposed to be modulated by phosphoinositide lipids^39^. Heterologously expressed TRPV2 is sensitive to phosphoinositide lipid levels in the membrane^17^. In turn, endogenously expressed endosomal TRPV2 may be modulated by a variety of phosphoinositides, which are abundant in intracellular membranes^14^,^39^,^40^,^41^,^42^,^43^. In addition TRPV2 activity may be regulated by post-translational modifications, as well as changes in channel expression and localization^14^.

Reconstituted full-length TRPV2 displays activity in the presence of ligand in artificial liposomes^16^. Additional patch clamp electrophysiology experiments revealed that pure TRPV2 exhibits long spontaneous opening events in the absence of ligands, whereas protein-free liposomes were silent (Supplementary Fig. 1d). This activity may be intrinsic to TRPV2; however, it remains possible that TRPV2 function is altered in the presence of cellular factors absent from the reconstituted system, such as membrane lipids that modulate channel activity^41^. The absence of cellular factors during cryo-EM data acquisition may also affect the overall full-length TRPV2 architecture in the apo state. Further studies are necessary to determine how endogenous regulators lead to conformational changes associated with activation of TRPV2.

From a technical point of view, cryo-EM is revolutionizing our ability to determine structures of membrane-spanning channels^20^,^44^. This study further highlights the power of this technology by resolving the structure of the full-length TRPV2. Additional structural analyses of TRPV2 in the presence of channel modulators, as well as other TRP channels will shed light on the diverse regulation mechanisms and functions of this large superfamily of ion channels.

## Methods

### Cell culture and transfection

F11 cell line was a kind gift from Sharona Gordon (the University of Washington). Plasmids encoding rat TRPV1 and TRPV2 for mammalian expression were a kind gift from David Julius (the University of California San Francisco). For protein expression, rat TRPV2 was cloned in frame with a C-terminal 1D4 epitope (TETSQVAPA) into the YepM plasmid^16^. For immunocytochemistry, rat TRPV1 and TRPV2 were separately cloned in frame with a C-terminal 1D4 epitope into the pcDNA3.1 plasmid^13^. The pore turret-truncated TRPV2 mutant construct with residues 568–589 deleted was created using mutated oligonucleotides (TRPV2-Δ564–589 Forward 5′-TTGAGCAGAGAGGCCCGATATCGGAGC-3′; Reverse 5′-ATCCAGAATGCTCCGATATCGGGCCTC-3′), WT TRPV2-1D4 in pcDNA3.1 as a template and Accuprime DNA polymerase kit (Life Technologies). pCAG-mGFP (plasma membrane-GFP, GFP-labelled palmitoylation sequence from GAP43) was a gift from Connie Cepko (Addgene plasmid #14757)^45^. F11 cells were cultured with Ham's F-12 nutrient mix (Invitrogen) supplemented with 10% foetal bovine serum (Cellgro), 1 × HAT supplement (Invitrogen) and 1% Penstrep (Invitrogen) and maintained in a humidified atmosphere at 37 °C and 5% CO_2_.

Transfections were performed as previously described^13^. In brief, plasmids were transfected using polyethyleneimine (PEI, Polysciences). A mixture of OPTIMEM (Invitrogen), 2.5 μg of DNA and 3 μg of PEI was incubated at room temperature for 10 min prior to adding the mixture to the cells for a 4-h incubation period at 37 °C. The cells were replaced with fresh growth media after the 4-h incubation and incubated for 48 h before harvesting or fixing.

### Immunocytochemistry

Immunocytochemistry was performed as described previously^13^. In brief, F11 cells were seeded onto 35 mm iBidi dishes overnight at 37 °C prior to transfection. After transfection, the cells were washed with Dulbecco's phosphate-buffered saline (DPBS, Invitrogen) containing CaCl_2_. The cells were fixed with 4% paraformaldehyde and blocked and permeabilized with PBS-0.3% Triton X-100 (PBS-T) containing 5% normal goat serum. Next, cells were incubated with Alexa 568-labelled 1D4 antibody for 1 h followed by three washes in DPBS. Images were obtained using a Leica TCS SP2 confocal microscope.

### Cell surface biotinylation

Cell surface biotinylation experiments were performed as described^13^. In short, F11 cells were transfected with either WT TRPV2 or TRPV2 Δ564–589. Surface-expressed proteins were biotinylated with sulfo-*N*-hydroxysuccinimide (NHS)–biotin (Thermo Scientific, Waltham, MA) for 30 min at 37 °C. Afterwards, the cells were quenched with 100 mM Glycine in PBS and lysed with *L*-RIPA buffer. Streptavidin–agarose beads (Life Technologies) were used to pull down biotinylated protein followed by western blotting analysis. WT TRPV2 and TRPV2 Δ564–589 were detected with 1D4 antibody (1:1,000), which was generated from our laboratory^46^. Na^+^/K^+^ ATPase (catalogue number 3010, 1:100, Cell Signaling Technology) and β-actin (catalogue number 3700, 1:1,000, Cell Signaling Technology) were used as surface biotinylation and loading controls, respectively.

### Cytosolic Ca^2+^ measurements

Ca^2+^ imaging experiments were performed with membrane-permeable Fluo4 (Fluo4-AM, Life Technologies) as described previously^14^. In short, F11 cells were co-transfected with the TRPV2 protein and red fluorescent protein (RFP), which identified transfected cells. The cells were loaded with 5 μM Fluo4-AM in extracellular solution (ECS) containing 20 mM HEPES (pH 7.3), 120 mM NaCl, 2 mM CaCl_2_, 3 mM KCl, 2 mM MgCl_2_, 20 mM glucose and 11 mM sorbitol. Following a 30-min incubation at room temperature with Fluo4-AM, the cells were washed twice with ECS before fluorescence measurements. Fluorescence measurements from single cells were taken using C1 Plus confocal system on a Nikon Eclipse Ti-E microscope (Nikon Instruments Inc., Melville, NY) at room temperature with excitation and emission wavelengths at 495 nm and 520 nm, respectively. Images were recorded at 3-s intervals for a total of 300 s. Fluorescence intensities were analysed using ImageJ software.

### Protein expression and purification

Rat TRPV2 with C-terminal 1D4 tag was expressed and purified using the same procedure described from our previous studies^16^. Protein was expressed in *Saccharomyces cerevisiae* and membranes were isolated after lysis. TRPV2 was solubilized in a buffer that consists of 0.87 mM lauryl MNG (LMNG, Anatrace), 150 mM NaCl, 20 mM HEPES (pH 8.0), 5% glycerol, 1.0 mM dithiothreitol (DTT) and 1.0 mM phenylmethylsulfonyl fluoride for 1 h. Insoluble membrane was pelleted after a 100,000*g* ultracentrifugation then incubated overnight with cyanogen bromide-activated-Sepharose 4B beads (GE healthcare) coupled with 1D4 antibody at 4 °C. The protein was purified through immuno-affinity chromatography, washed with buffer containing 0.064 mM decyl MNG (DMNG, Anatrace), 150 mM NaCl, 20 mM HEPES (pH 8.0) and 1.0 mM DTT and eluted with 3.0 mg ml^−1^ of 1D4 peptide in wash buffer. The affinity-purified protein was then further purified by size-exclusion chromatography on a Superose 6 Column (GE healthcare). The purified TRPV2 fractions were collected and concentrated with 100 kDa molecular weight cut-off concentrators (GE healthcare) to ∼2.5 mg ml^−1^.

### Electrophysiology recordings

All electrophysiological measurements were made by patch clamp recordings in reconstituted proteoliposomes, as described earlier^16^,^47^,^48^. A total of 20 mg soybean polar extract (Avanti polar lipids) in chloroform was dried in nitrogen stream and sonicated in a buffer containing 200 mM KCl and 10 mM 3-(*N*-morpholino)propanesulfonic acid (MOPS) at pH 7.0. The liposomes (at 10 mg ml^−1^ concentration) were destabilized by adding 4 mM DDM and were gently agitated for 20 min. Purified TRPV2 was added to the vesicles (in 1:50 (w/w) protein to lipid ratio) and nutated for upto 2 h. Residual detergent was removed by incubation with biobeads (Bio-Rad Laboratories) overnight at 4 °C. Channel-incorporated liposome suspension was then centrifuged for 2 h at 100,000*g* and the pellet was resuspended in 60 ml of KCl/MOPS buffer. A drop of the proteoliposome preparation was placed on a glass slide and dried overnight in a desiccator at 4 °C. The sample was then rehydrated with 0.5 ml of buffer, which yielded giant liposomes. This preparation was suitable for patch clamp recordings after 2 h. All measurements in this study were conducted in the inside-out patch configuration with a bath solution containing 150 mM KCl, 10 mM MOPS and pH 7.0 at room temperature (21 °C). Single currents were acquired at 10 kHz and filtered at 2 kHz using Axon 200-B patch-clamp amplifier (Molecular Devices).

### Cryo-EM data collection

Purified rat TRPV2 was frozen using conditions as previously described^16^. Briefly, a 3.0 μl aliquot of purified protein at a concentration of 2.5 mg ml^−1^ was blotted onto Quantifoil R2/1 grids (Quantifoil Micro Tools) and manually plunged into liquid ethane. The frozen grid samples were imaged on the FEI Titan Krios Microscope operated at 300 kV at the nominal magnification of × 31,000 with a Gatan K2 Summit direct detector camera. Movies (32 frames per movie) were recorded under super-resolution counting mode at a calibrated pixel size of 0.645 Å and a dose rate of ∼8 electrons per physical pixel per s and underfocus values ranging from −1.5 to −3.0 μm with the automated collection software Leginon^49^.

### Image processing

Sub-frames 3 through 16 from the image stack were motion corrected, binned by 2 and averaged using the motioncorr program^50^. Particle auto-picking procedure was performed in RELION 1.3 (ref. 21). From 988 movies, 1,009 particles were manually picked followed by 2D classification. Representative views from the 2D classes were chosen as templates for auto-picking procedure that resulted in 218,897 particles picked. Classification, refinement and movie processing were performed using RELION 1.4 (ref. 21). The auto-picked particles underwent 25 iterations of reference-free 2D classification. The best 2D classes containing a total of 106,193 particles were chosen for 25 iterations of unsupervised 3D classification generating 6 classes using the 13.6 Å TRPV2 map^16^ as the initial model. 3D auto-refinement was performed using 42,550 particles from the best 3D class and the corresponding map low-passed filtered to 60 Å as the initial model. The 4.6 Å map was generated after post-processing with RELION 1.4 auto-masking procedure with an initial binarization threshold of 0.008, a binary map extension by 1 pixel and an addition of 6 pixels for soft-edge masking. The map was sharpened with a bfactor of −226 Å^2^ determined by auto-bfactor procedure during post-processing. To further improve the 3D map, particle polishing was performed with the 42,550 particles^51^. Particles were extracted from all 32 frames and polished with running average frames of 7. A 25 iteration round of 3D auto-refinement was performed following particle polishing with the ‘shiny' particle data set. Afterwards, post-processing was performed applying auto-masking with an initial binarization threshold of 0.016, a binary map extension by 6 pixels and an addition of 5 pixels for soft-edge masking. The map was sharpened with a bfactor of −157 Å^2^ determined by auto-bfactor procedure. The resolution of the TRPV2 structure ranges from ∼4.5 Å (FSC 0.143) to ∼5.5 Å (FSC 0.5). Local resolution was evaluated using the post-processed map with ResMap^52^.

### TRPV2 model building and refinement

An initial homology model was generated with the I-TASSER^53^ server employing the three previous structures of TRPV1 (apo-TRPV1 PDB: 3J5P, capsaicin–TRPV1 PDB: 3J5R, RTX/DkTx–TRPV1 PDB: 3J5Q), as well as the high-resolution structure of the TRPV2 ARD (PDB: 2ETB). This initial model was aligned to our TRPV2 cryo-EM map calculated with RELION 1.3 and was manually adjusted to fit the electron density in COOT^54^. After this initial model fitting, we refined this model against the EM-derived maps using real space refinement (RSR) as implemented in the PHENIX software package^55^. During the review process, we recalculated maps utilizing RELION 1.4, which resulted in considerably better map connectivity and completeness and enabled us to further improve our initial model. After this initial model fitting, we refined this model against the EM-derived maps using the RSR and simulated annealing and local grid minimization as implemented in the PHENIX software package^55^. The cryo-EM structures of TRPV1 (PDB: 3J5P, 3J5R, 3J5Q) and rabbit TRPV2 (PDB: 5AN8) were used as references during manual building and structure adjustment, and assisted in assignment of topology and improved connectivity in the model. The model was subjected to additional rounds of manual model fitting and RSR, resulting in a final model to map cross-correlation coefficient of 0.706. The TRPV2 model was superimposed to TRPV1 models using the superpose command in CCP4 suite.

### Masked 3D classification with signal subtraction

Masked 3D classification procedure^56^ was performed using RELION 1.4 to improve the densities in the more flexible regions of our full-length TRPV2 structure. A map of the TM region with the MNG detergent belt was generated using the TRPV2 refined model of the TRPV2 cytoplasmic domains and the split map feature in UCSF Chimera. The map was then converted to a soft-mask using the relion_mask_create command. The soft-mask of the TM region was subtracted from the soft-mask generated from the final post-processing procedure using the subtract option from the relion_image-handler command to generate a soft-mask of the cytoplasmic region. A consensus 3D auto-refinement was performed using the cytoplasmic region soft-mask and the 42,550 particles that generated the∼4.5 Å (FSC 0.143) map to generate a refined map of the cytoplasmic region. The map was employed as a template for signal subtraction from the particles. The final particle stack with the subtracted signal was created using the relion_project and was implemented in 3D classification. From the 4 3D classes, the non-subtracted particles from 2 3D classes with a total of 29,765 particles were selected for final 3D auto-refinement. Post-processing was performed applying auto-masking with an initial binarization threshold of 0.008, a binary map extension by 3 pixels and an addition of 5 pixels for soft-edge masking. The map was sharpened with a bfactor of −133 Å^2^ determined by auto-bfactor procedure. The resolution of this TRPV2 structure ranges from ∼4.8 Å (FSC 0.143) to ∼5.5 Å (FSC 0.5). Although the overall resolution of the map is lower, the densities in the TM region have improved. The model was adjusted in this region to fit into the density and refined with RSR.

## Additional information

**Accession codes:** The final cryo-EM density map and the signal subtracted classified density map have been deposited in the Electron Microscopy Data Bank, under accession numbers EMD-6580 and EMD-6618 respectively. Atomic model has been deposited in the Protein Data Bank under accession code 5HI9.

**How to cite this article:** Huynh, K. W. *et al*. Structure of the full-length TRPV2 channel by cryo-EM. *Nat. Commun.* 7:11130 doi: 10.1038/ncomms11130 (2016).

## Supplementary Material

Supplementary Information Supplementary Figures 1-9

Supplementary Movie 1 Rotation of the S1 domain of TRPV2. The model of the TRPV2 S1 domain superimposed onto the cryo-EM densities, as in related to Fig 1d.

Supplementary Movie 2 Rotation of the β-strand region of TRPV2. The model of the β- strand region of TRPV2 superimposed onto the cryo-EM densities, as in related to Fig 1d.
