# Supplementary material for: Structure of the full-length TRPV2 channel by cryo-EM
Source: Nat Commun. 2016 Mar 29;7:11130. doi: 10.1038/ncomms11130 (PMC4820614; doi:10.1038/ncomms11130)
Supplement: Supplementary Information — Supplementary Figures 1-9 [file ncomms11130-s1.pdf]

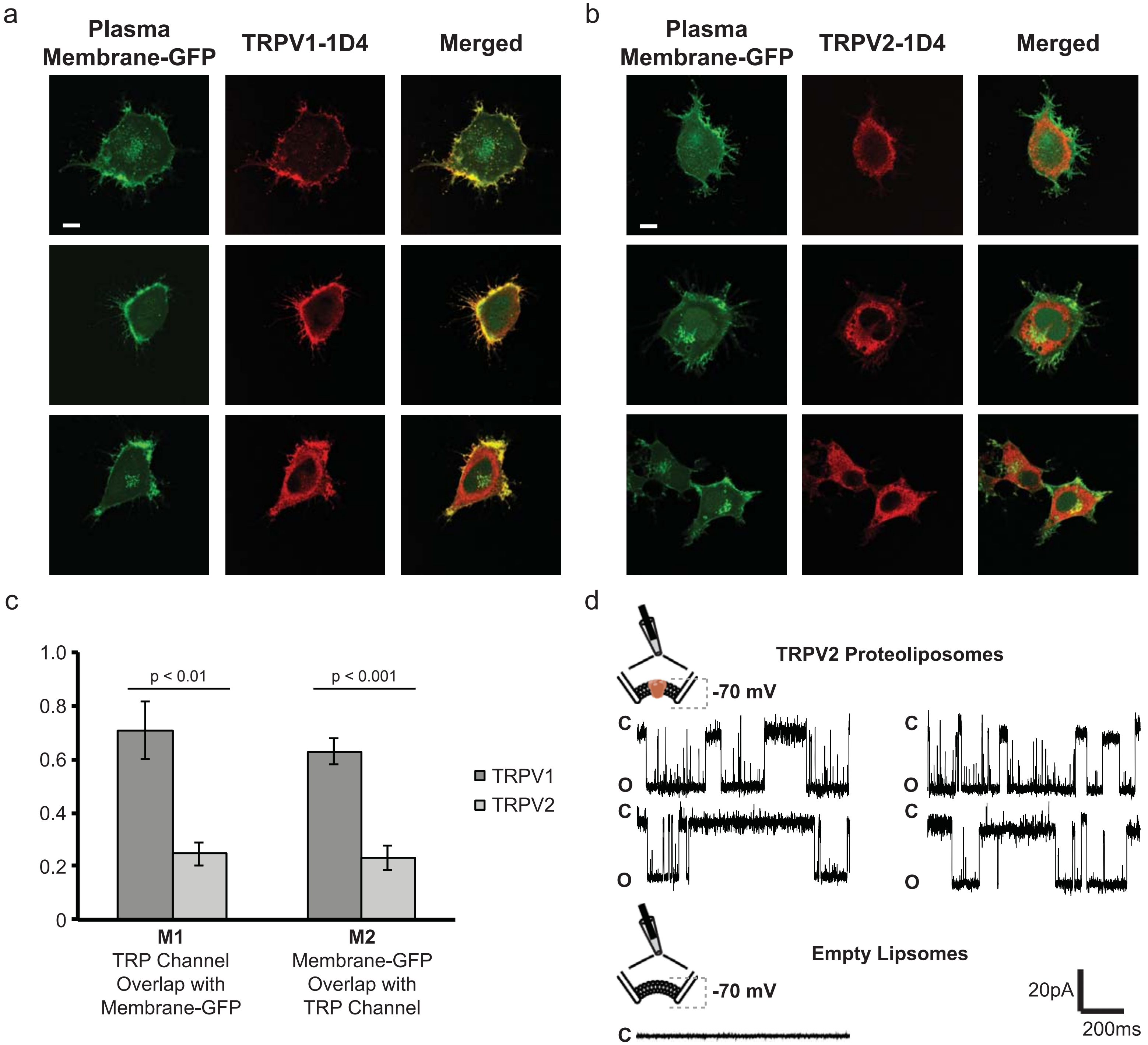

Supplementary Fig. 1 | Functional characterization of TRPV2. F11 cells were co-transfected with plasma membrane-GFP (GFP-labeled palmitoylation sequence from GAP43; green) and either **(a)** TRPV1-1D4 or **(b)** TRPV2-1D4. Cells were fixed and immunostained using anti-1D4 antibody (red). Scale bar represents 10 µm. **(c)** Colocalization analysis for images represented in (a) and (b). Mander's colocalization coeffecients were determined using the JACoP plugin for ImageJ52. M1 represents TRPV1-1D4 (n=5) or TRPV2-1D4 overlap with mGFP (n=8); M2 represents overlap of mGFP with TRPV1-1D4 or TRPV2-1D4. Costes' automatic thresholding was applied. Data represent mean ± SEM. **(d)** Excised inside-out patches of TRPV2-containing proteoliposomes and empty liposomes. Single-channel recordings were performed with symmetrical concentrations of KCl (150mM) in the pipette and in the bath solution. The membrane potential was held at -70mV.

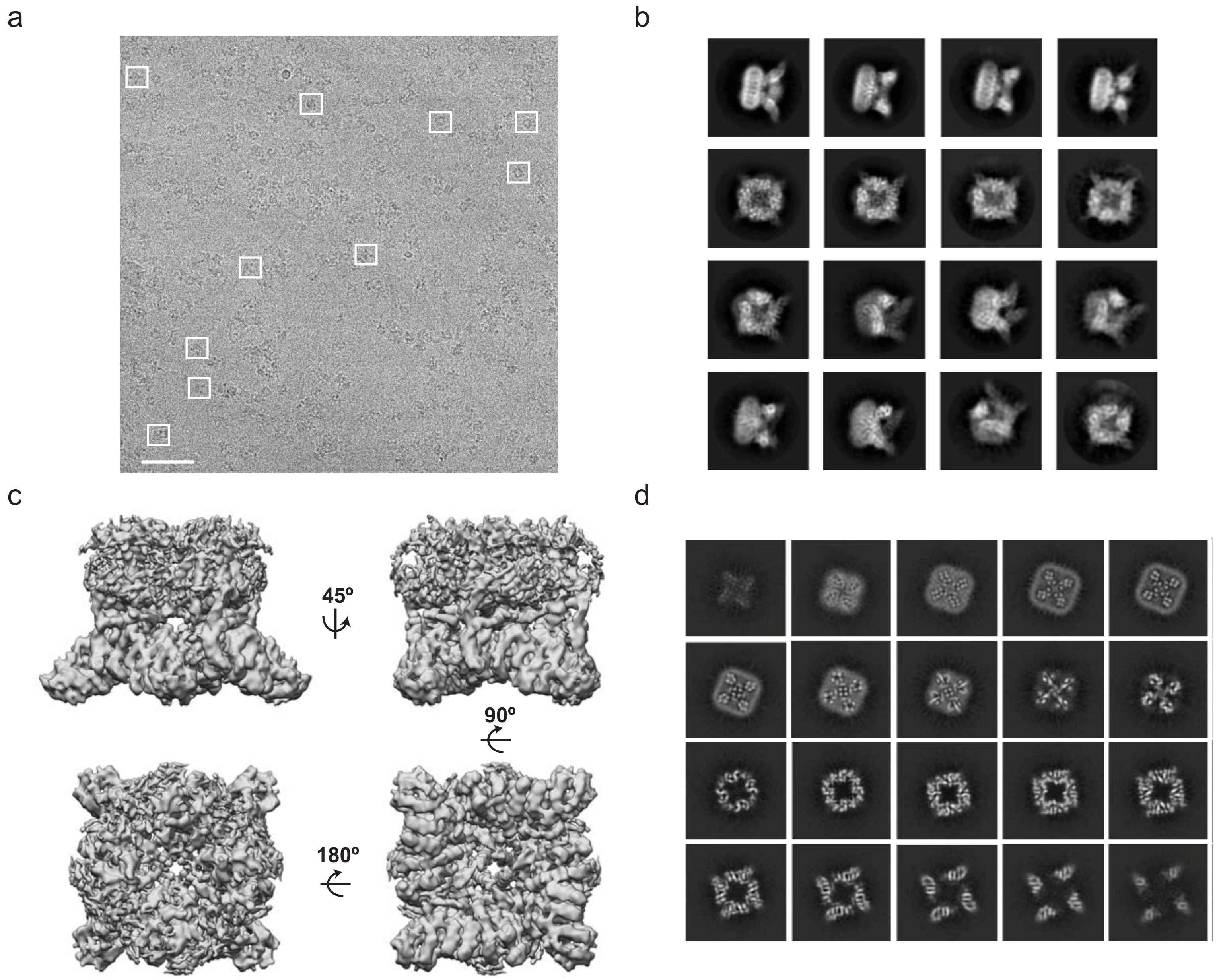

Supplementary Fig. 2 | 3D reconstruction of full-length TRPV2 **(a)** A representative micrograph obtained by aligning and averaging subframes 3-16 of a movie stack. White boxes indicate individual particles. Scale bar represents 50nm. **(b)** Representative 2D class averages of the TRPV2 cryoEM particles. Details of the channel subunits are visible from these 2D class averages. **(c)** Final 3D cryoEM map shown in four different views. **(d)** Slices along the pore axis of the final reconstruction starting from the extracellular side.

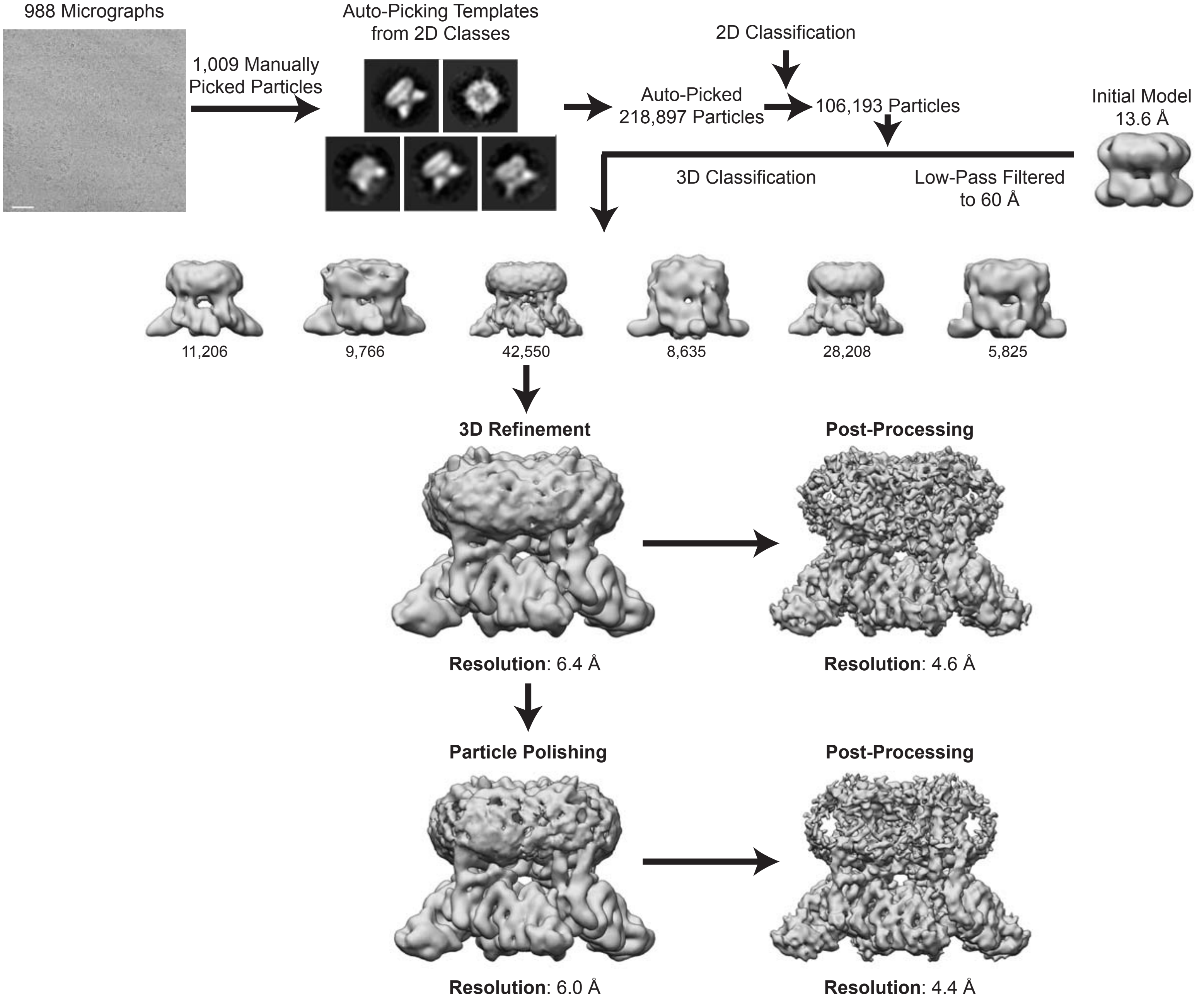

Supplementary Fig 3 | Schematic flow chart of the classification and refinement procedures to resolve the full-length TRPV2 cryoEM structure. From 988 micrographs, 218,897 particles were auto-picked and classified. 106,193 particles underwent a round of 3D classifications using the 13.6 Å TRPV2 cryoEM map17 low-passed filter to 60 Å as the initial model. 3D refinement performed on the 42,550 particles after 3D classification resulted in a 4.6 Å structure. Particle polishing slightly improved the reconstruction to 4.4 Å. The auto-picking procedure was performed in RELION 1.3. The classification, refinement and particle polishing steps were performed with RELION 1.4.

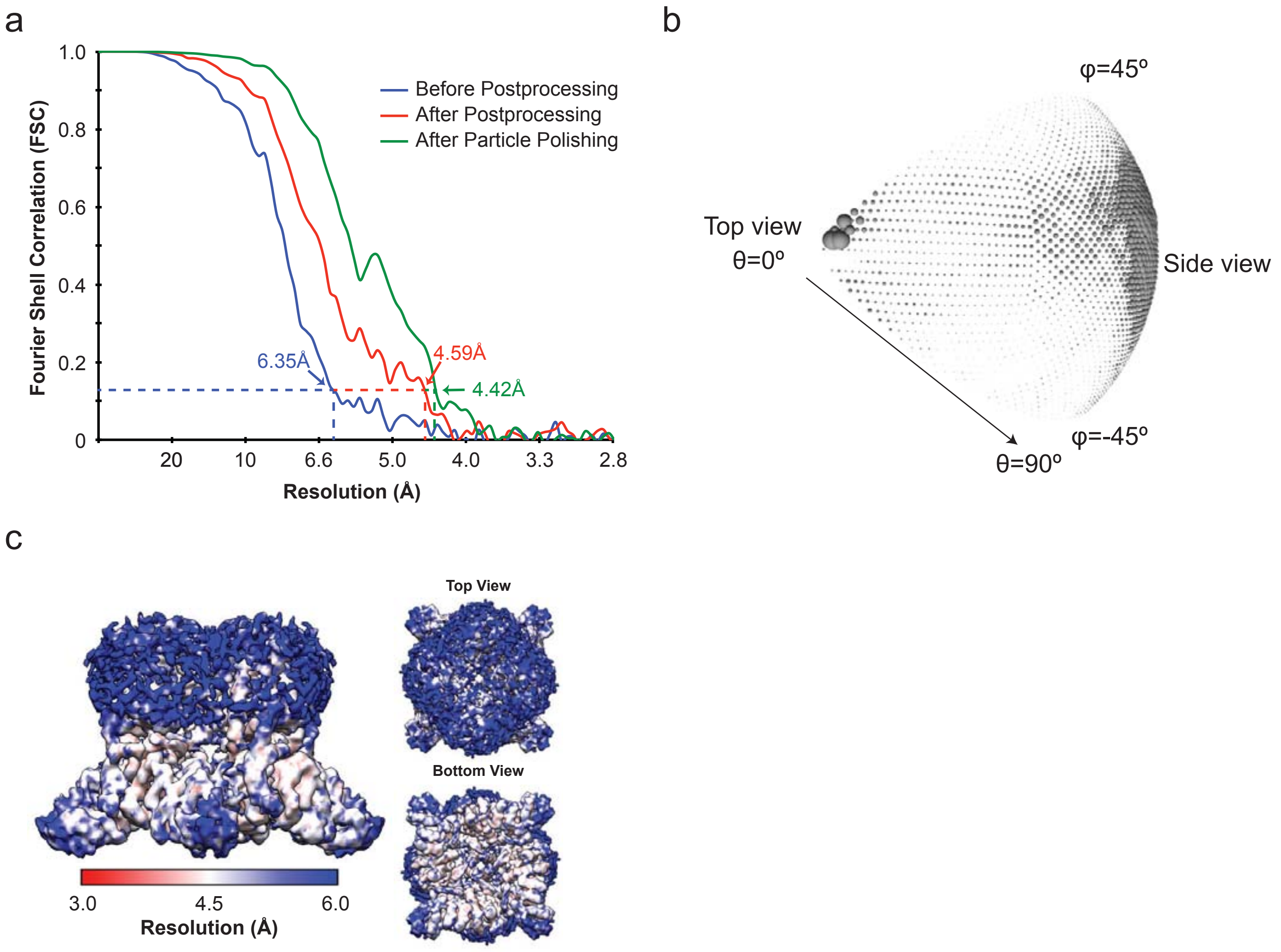

Supplementary Fig. 4 | Resolution analyses of TRPV2 cryoEM density. (a) Fourier shell correlation (FSC) between the TRPV2 cryoEM maps prior to RELION post-processing (blue), following RELION post-processing (red) and following RELION particle polishing (green). Resolution was determined using the FSC = 0.143 criterion of the post-processed TRPV2 map. **(b)** The Euler angle distribution plot for all 42,550 particles that were refined for the final model. **(c)** Local resolution analysis of the final TRPV2 cryoEM map performed by ResMap.

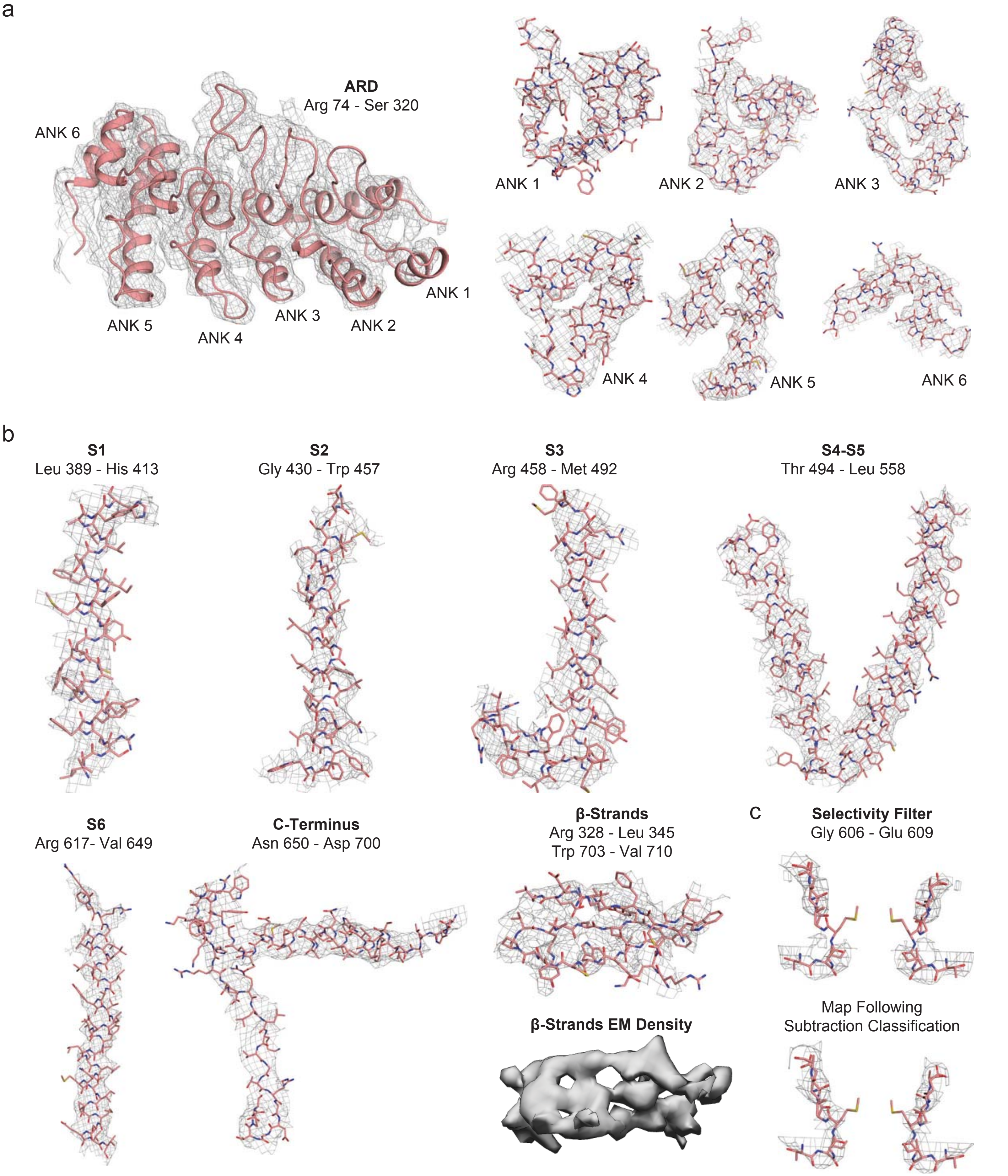

Supplementary Fig. 5 | CryoEM densities of selected regions of TRPV2. Representative cryo-EM densities are superimposed onto the atomic model for **(a)** the ankyrin repeat domain and **(b)** various TRPV2 domains as indicated. **(c)** Cryo-EM densities of the selectivity filter region prior to (top) and following (bottom) 3D classification with signal subtraction.

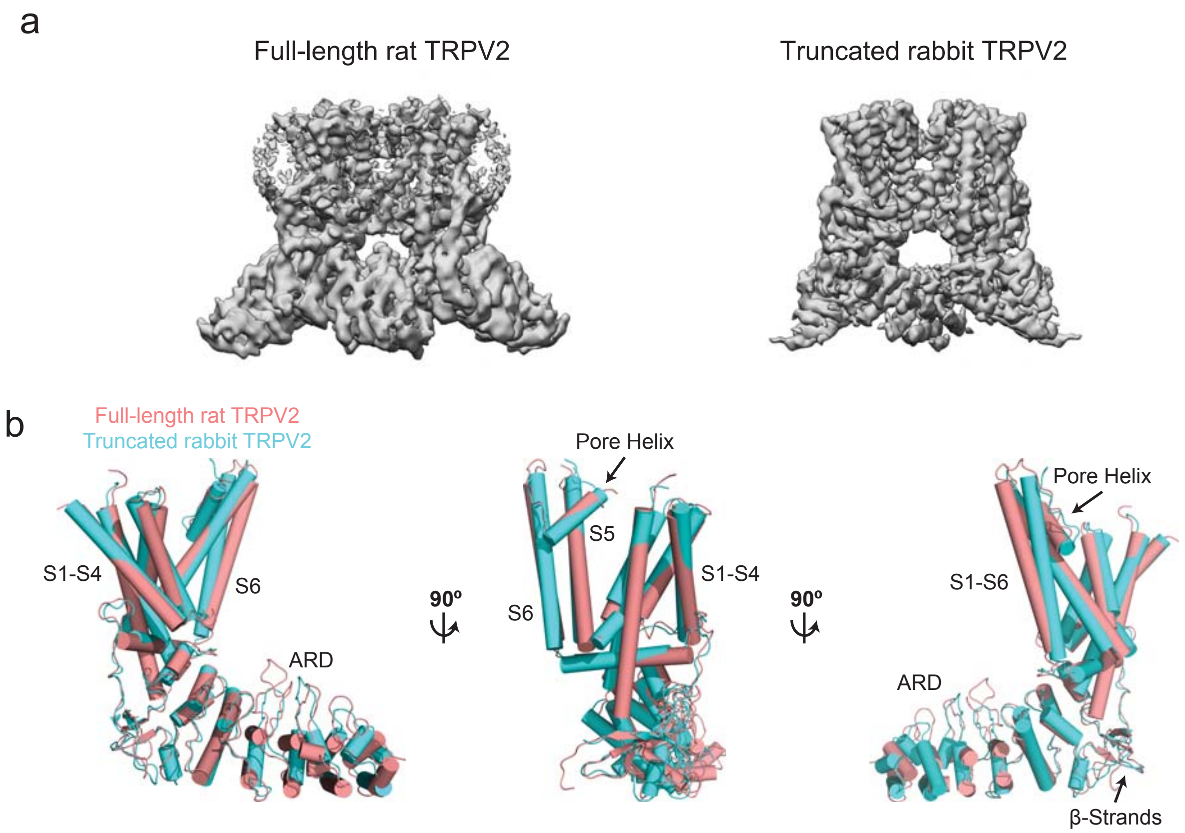

Supplementary Fig. 6 | Full-length and truncated TRPV2 structure comparison. (a) The cryo-EM densities of full-length rat TRPV2 and truncated rabbit TRPV2. **(b)** The models of a monomer of full-length rat TRPV2 (salmon) and truncated rabbit TRPV2 (cyan) superimposed. α-helices are represented as cylinders.

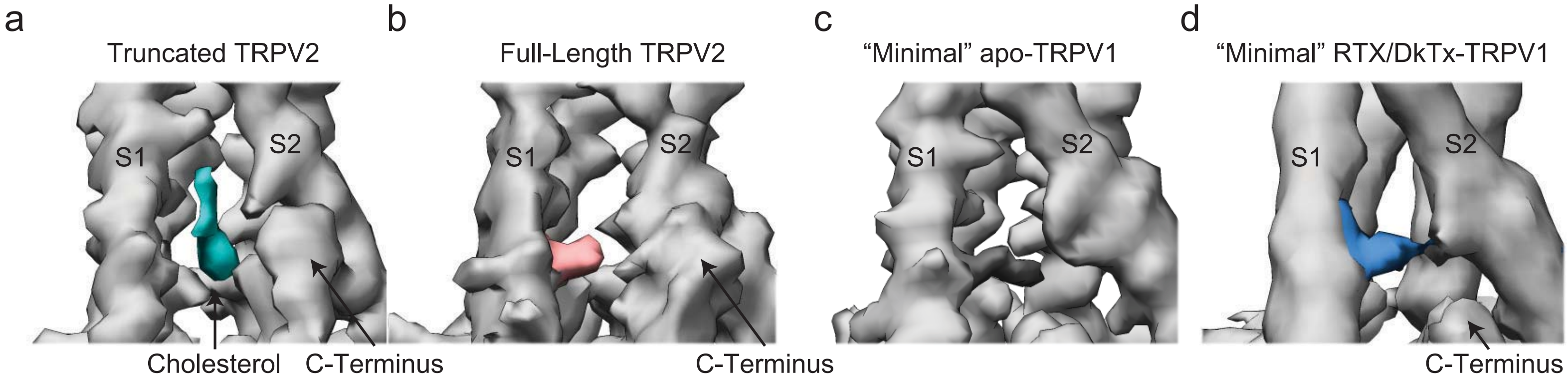

Supplementary Fig. 7 | Comparison of the lipid binding site densities among the TRPV cryo-EM stuctures. Cryo-EM densities of the lipid binding site located between the transmembrane S1-S4 helices bundle above the TRP domain from **(a)** truncated rabbit TRPV2, **(b)** full-length rat TRPV2, **(c)** “minimal” apo-TRPV1 and **(d)** “minimal” RTX/DkTx-TRPV1. The proposed cholesterol molecule density in the truncated rabbit TRPV2 is represented in cyan. The salmon, dark gray and blue colored densities are similar region densities in full-length rat TRPV2, “minimal” rat apo-TRPV1 and “minimal” rat RTX/DkTx-TRPV1 respectively.

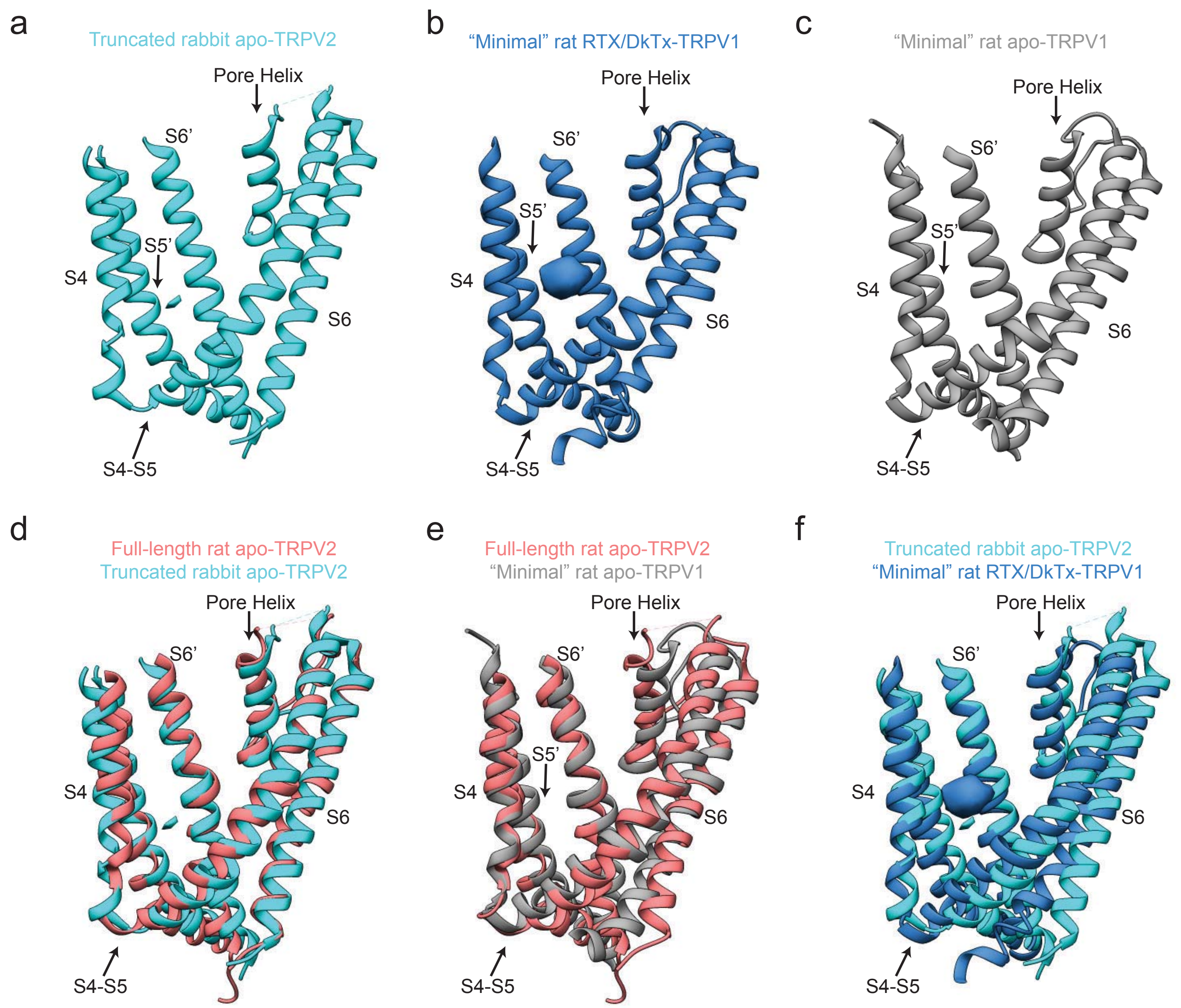

Supplementary Fig. 8 | Structural analysis of the vanilloid pocket among the TRPV structures. Models of **(a)** truncated rabbit TRPV2, **(b)** "minimal" rat RTX/DkTx-TRPV1 and **(c)** "minimal" rat apo-TRPV1 at the vanilloid pocket. The densities of the proposed lipid (cyan) and resiniferatoxin (blue) molecules are represented at the suggested contour level with the models. The lipid molecule in the "minimal" rat apo-TRPV1 is not apparent at the suggested contour level. **(d)** Superimposed full-length rat TRPV2 and truncated rabbit TRPV2 shown at the vanilloid pocket. **(e)** Superimposed models of full-length rat TRPV2 and "minimal" rat apo-TRPV1 shown at the vanilloid pocket. **(f)** Superimposed models of truncated rabbit TRPV2 and "minimal" rat RTX/DkTx-TRPV1 shown at the vanilloid pocket.

a

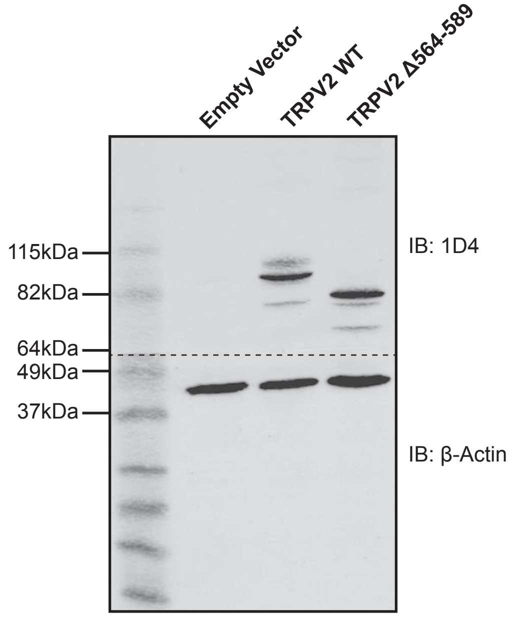

b

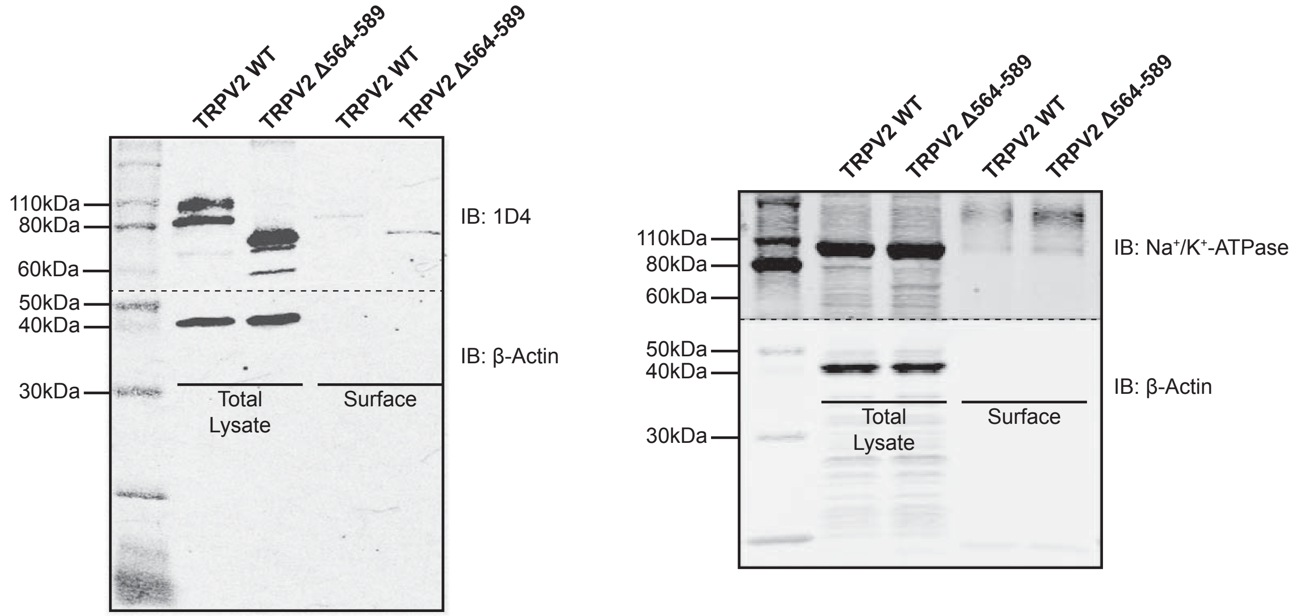

Supplementary Fig. 9 | Full immunoblots for Figures 4b and 4d. (a) Full immunoblot with indicated antibodies for F11 cells expressing empty vector, TRPV2 WT or TRPV2 Δ564-589 (see Fig. 4b). **(b)** Full immunoblots with indciated antibodes from cell surface biotinylation assay for F11 cells expressing TRPV2 WT or TRPV2 Δ564-589 (see Fig. 4d).
